# Chandelier Cells in Functional and Dysfunctional Neural Circuits

**DOI:** 10.3389/fncir.2016.00033

**Published:** 2016-05-04

**Authors:** Yiqing Wang, Peng Zhang, Daniel R. Wyskiel

**Affiliations:** ^1^Department of Pharmacology, University of VirginiaCharlottesville, VA, USA; ^2^Department of Chemistry, University of VirginiaCharlottesville, VA, USA

**Keywords:** chandelier cells, axo-axonic cells, interneuron, circuits, schizophrenia, epilepsy

## Abstract

Chandelier cells (ChCs; also called axo-axonic cells) are a specialized GABAergic interneuron subtype that selectively innervates pyramidal neurons at the axon initial segment (AIS), the site of action potential generation. ChC connectivity allows for powerful yet precise modulation of large populations of pyramidal cells, suggesting ChCs have a critical role in brain functions. Dysfunctions in ChC connectivity are associated with brain disorders such as epilepsy and schizophrenia; however, whether this is causative, contributory or compensatory is not known. A likely stumbling block toward mechanistic discoveries and uncovering potential therapeutic targets is the apparent lack of rudimentary understanding of ChCs. For example, whether cortical ChCs are inhibitory or excitatory remains unresolved, and thus whether altered ChC activity results in altered inhibition or excitation is not clear. Recent studies have shed some light onto this excitation-inhibition controversy. In addition, new findings have identified preferential cell-type connectivities established by cortical ChCs, greatly expanding our understanding of the role of ChCs in the cortical microcircuit. Here we aim to bring more attention to ChC connectivity to better understand its role in neural circuits, address whether ChCs are inhibitory or excitatory in light of recent findings and discuss ChC dysfunctions in brain disorders.

## Introduction

Discovered in the 1970s, ChCs quickly gained intrigue as a unique and potentially powerful subtype of GABAergic interneurons that selectively innervates pyramidal neurons at the axon initial segment (AIS), directly regulating the site of action potential generation (Szentágothai and Arbib, 1974; Jones, 1975; Somogyi, 1977). In the decades that followed, many studies have uncovered potential roles of ChCs in brain functions (Li et al., 1992; Klausberger et al., 2003; Zhu et al., 2004; Howard et al., 2005; Dugladze et al., 2012; Jiang et al., 2013; Viney et al., 2013), and have led to implications of ChC dysfunctions in brain disorders such as epilepsy and schizophrenia (DeFelipe, 1999; Lewis, 2011; Marín, 2012; Inan and Anderson, 2014). However, many questions remain unanswered. One of the most puzzling questions involves whether these GABAergic interneurons can be excitatory (Szabadics et al., 2006; Woodruff et al., 2010). In addition, how ChCs are incorporated in neuronal circuits is not clear. The diseases that implicate ChC dysfunction also involve other cell types, including other interneuron subtypes (DeFelipe, 1999; Lewis et al., 2012; Del Pino et al., 2013). Recent studies have shed some light onto the excitation-inhibition controversy and ChC connectivity in the cortex, which may facilitate our understanding of ChC functions in neural circuits and ChC connectivity dysfunctions in brain disorders. Here we briefly review ChCs in functional and dysfunctional neural circuits and highlight these new findings.

## ChC Connectivity

**Figure 1:**
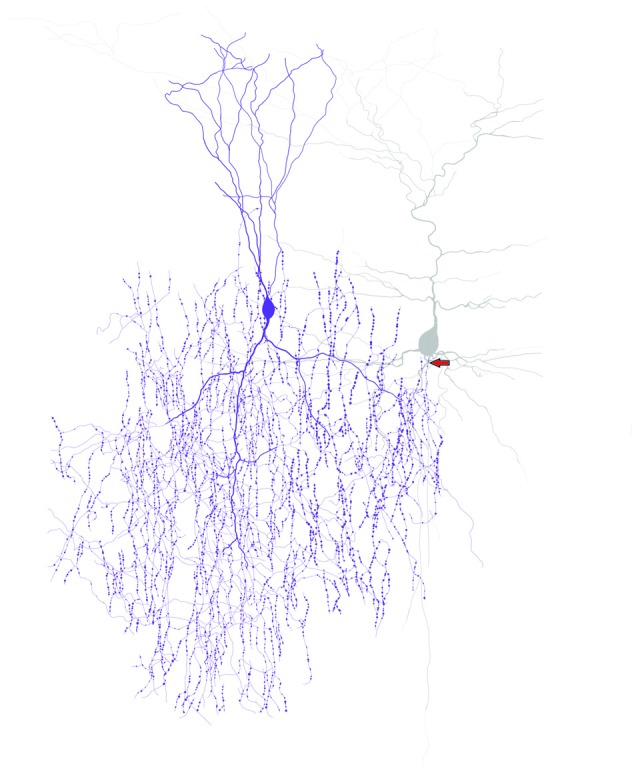
**ChCs have unique axonal morphology and innervate pyramidal neurons at the axon initial segment (AIS).** Drawing of a ChC (purple) and a connected pyramidal neuron (gray) to illustrate the “chandelier” morphology and the axo-axonic connectivity of ChCs. Arrow indicates the ChC connectivity site at the pyramidal neuron AIS.

ChC connectivity to the AIS of pyramidal neurons has been found in many different brain regions of many different animals, including the human prefrontal cortex (Somogyi, 1977; Fairén and Valverde, 1980; Peters et al., 1982; Somogyi et al., 1982, 1983; Freund et al., 1983; Kosaka, 1983; DeFelipe et al., 1985; Kisvárday et al., 1986; De Carlos et al., 1987; Marin-Padilla, 1987; Lewis and Lund, 1990; Kawaguchi and Kubota, 1998; Inda et al., 2007). The axonal arborization of a ChC forms vertically oriented axon terminal boutons or cartridges, a distinct arrangement resembling candlesticks on a chandelier (Szentágothai and Arbib, 1974; Jones, 1975; Szentágothai, 1975; see Figure 1). ChC cartridges align with AISs of pyramidal neurons, allowing a single ChC to innervate the AIS with an average of 3–5 boutons. This innervation average is highly variable. The number of ChC boutons per AIS is not uniform across brain regions, is directly correlated with the size of the pyramidal AIS and may reach as many as 12 (DeFelipe et al., 1985; Fariñas and DeFelipe, 1991; Cruz et al., 2003; Inda et al., 2007; Inan and Anderson, 2014). In addition, this mean value is variable during development, as the number of ChC boutons per AIS is 32% lower in adult compared to 3-month-old monkeys (Fish et al., 2013).

A single ChC innervates hundreds of pyramidal neurons (Freund et al., 1983; DeFelipe et al., 1985; Somogyi et al., 1985; Li et al., 1992; Tai et al., 2014). Within the range of its axonal arbor, a ChC contacts 35–50% of pyramidal neurons in the somatosensory cortex through postnatal development (Inan et al., 2013); however, lower innervation values (18–22%) by single ChCs have been reported when examining a wider area of the neocortex in postnatal day 18–23 (P18–23) mice (Blazquez-Llorca et al., 2015). Quantitative analysis showed that this connectivity reaches a peak of 22–35% at 30–60 μm from the ChC soma (Blazquez-Llorca et al., 2015). The selective innervation at the AIS suggests that ChCs tightly regulate the output of pyramidal neurons. Moreover, each pyramidal neuron is innervated by multiple ChCs (Inan et al., 2013). As the number of functional release sites critically regulates the firing probability (Loebel et al., 2009; Bagnall et al., 2011), multiple innervations further contribute to the ability of ChCs to strongly and precisely regulate pyramidal neurons (Buhl et al., 1994).

ChCs innervate pyramidal neurons in cortical layer 2 (L2), L3, L5a and L5b (Jiang et al., 2013; Lee et al., 2015). This innervation of cortical pyramidal neurons shows clustered patterns of both high and very low densities based on the identification of ChC cartridges and their apposition to AISs (Fairén and Valverde, 1980; Somogyi et al., 1982; DeFelipe et al., 1985; Li et al., 1992; Inan et al., 2013; Blazquez-Llorca et al., 2015). This may be due to differences in ChC morphology or distribution as ChCs are not distributed uniformly in certain areas of the cortex (De Carlos et al., 1985). Another possibility is that ChCs may preferentially target certain neuronal groups over others. Although the connectivity between GABAergic interneurons and pyramidal neurons has been hypothesized to be generally non-selective and based primarily on spatial proximity (Sohya et al., 2007; Niell and Stryker, 2008; Liu et al., 2009; Bock et al., 2011; Fino and Yuste, 2011; Packer et al., 2013), this may be greatly overstated (Varga et al., 2010). Indeed, evidence shows that ChCs preferentially contact certain pyramidal neurons over others, such as pyramidal neurons with predominantly intracortical projections in the auditory and visual cortices, and centrifugal cells in the piriform cortex (Sloper and Powell, 1979; Fairén and Valverde, 1980; De Carlos et al., 1985; DeFelipe et al., 1985; Fariñas and DeFelipe, 1991; Wang and Sun, 2012).

**Figure 2:**
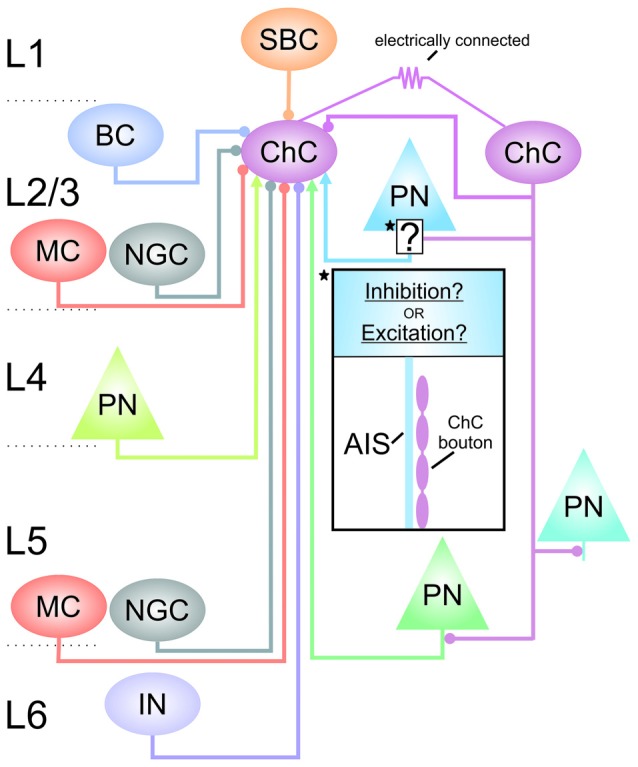
**Connectivity of cortical ChCs.** Simplified schematic of some of the known connections between L2/3 ChCs and other neurons in the cortex. Arrows at terminals indicate excitation; circles at terminals indicate inhibition. Starred and boxed region and insert highlights the ChC synaptic contact with L2/3 pyramidal neurons in which the GABAergic response is not clear. AIS, axon initial segment; BC, basket cell; ChC, chandelier cell; IN, interneuron of unknown subtype; MC, Martinotti cell; NGC, neurogliaform cell; PN, pyramidal neuron; SBC, single-bouquet cell.

Studies examining the inputs to L2/3 ChCs have elucidated some ChC cortical connectivity (see Figure 2) and offered some possible functional roles. Using laser scanning photostimulation, L2/3 ChCs in the mouse primary somatosensory cortex were shown to receive excitatory input predominantly from L2/3 and L5a (with relatively weaker excitatory input from L4), and receive inhibitory input primarily from L1 and L2/3 (with relatively weaker input from L5b and L6; Xu and Callaway, 2009). Dendrites from L2/3 ChCs extend branches within the lamina and send a prominent dendrite upward into L1 (Kawaguchi, 1995; Xu and Callaway, 2009; Woodruff et al., 2011; Taniguchi et al., 2013; Markram et al., 2015). These findings led to the hypothesis that the dendrites of L2/3 ChCs, similar to the apical dendrites of pyramidal neurons, allow ChCs to act as circuit switches by receiving input from other cortical areas via L1. Interestingly, dual recordings from both pyramidal neurons and ChCs in L2/3, revealed that the L1 stimulation strength necessary for activation is significantly less for ChCs compared to pyramidal neurons (Woodruff et al., 2011). Much insight into L2/3 ChC function has come from the examination of inputs with *in vivo* recordings. Using whisker stimulation, Zhu et al. (2004) demonstrated that L2/3 ChCs have large receptive fields with lower acuity than pyramidal neurons and other non-pyramidal neurons. In addition, simultaneous dual recordings *in vivo* showed that L2/3 ChCs respond more robustly to increased cortical excitation than other cortical neurons. These results suggest that L2/3 ChCs have a critical role in balancing excitation and inhibition (Zhu et al., 2004).

Recent studies have greatly advanced our current understanding of ChC cortical connectivity with GABAergic interneurons (see Figure 2). The L1 inhibitory input to L2/3 ChCs is now known to come from single-bouquet cells (SBCs; Jiang et al., 2013). Within L2/3, ChCs receive GABAergic inputs from Martinotti cells (MCs), neurogliaform cells (NGCs) and from other ChCs, along with some inputs from basket cells (BCs; Jiang et al., 2015). L2/3 ChCs also receive input from MCs and NGCs from L5 (Jiang et al., 2015). In addition, ChC connectivity through gap junctions has been reported in the hippocampus (Baude et al., 2007), and in the neocortex between ChCs and between ChCs and BCs (Woodruff et al., 2011; Taniguchi et al., 2013). These connectivity patterns may allow for the coordination of ChCs to synchronize the activity of large populations of pyramidal cells (Bennett and Zukin, 2004; Howard et al., 2005).

ChCs are diverse. Along with the diversity in the number of boutons (see above), ChC axons can vary in their complexity and localization in different cortical areas and layers, and depends on the type and age of the animal (Somogyi et al., 1982; DeFelipe et al., 1985; Inda et al., 2007, 2009; Taniguchi et al., 2013). Some genetic markers are thought to be specific for ChCs, such as DOCK7, a molecule essential for ChC cartridge and bouton development (Tai et al., 2014). However, ChCs show some diversity in their biochemical content. For example, studies indicate that only subpopulations of ChCs express certain gene products used as markers for interneuron subtypes, such as parvalbumin (Lewis and Lund, 1990; Del Río and DeFelipe, 1994; Fish et al., 2013; Taniguchi et al., 2013). This diversity in ChCs suggests complex connectivities and possibly distinct functional roles. ChCs originate during the latest stages of cortical neurogenesis and then migrate through defined routes that lead to a specific laminar distribution in the cortex (Inan et al., 2012; Taniguchi et al., 2013). This laminar distribution of ChCs is established prior to their innervation of pyramidal neurons and has led to the hypothesis that ChCs may be composed of different layer-specific subgroups, which establish distinct connectivities and perhaps distinct functional roles in the cortical microcircuit (Taniguchi et al., 2013).

## Are ChCs Inhibitory Or Excitatory?

Whether ChCs are inhibitory or excitatory is not currently agreed upon. ChCs activate GABA_A_ receptors and a greater understanding of the response mediated by these receptors is needed. Activation of GABA_A_ receptors in mature neurons is typically associated with inhibition owing to the flow of anions such as Cl^−^ through the membrane, leaving the membrane potential below threshold (Kaila, 1994). However, GABA_A_ receptors are capable of mediating excitation if the transmembrane gradient of Cl^−^ is reversed (Misgeld et al., 1986). GABA-mediated excitation is thought to occur in developing neurons until around P7 due to an intercellular Cl^−^ regulation that results in the efflux of Cl^−^, which raises the membrane potential above threshold (Obata et al., 1978; Mueller et al., 1984; Ben-Ari et al., 1989; Cherubini et al., 1991; Owens et al., 1996; Rivera et al., 1999; Ben-Ari, 2002; Owens and Kriegstein, 2002). Because intracellular Cl^−^ homeostasis may be altered by experimental procedures, these observations have been questioned (Bregestovski and Bernard, 2012; Dzhala et al., 2012; but see Ben-Ari et al., 2012b). Recently, *in vivo* recordings have demonstrated that GABA generally depolarizes but inhibits postsynaptic neurons in developing (P3–4) mice (Kirmse et al., 2015). However, concerns about the results of this study have been raised due to experimental procedures that can alter the recorded GABAergic activity (Ben-Ari, 2015). Thus, the effect of GABA observed is highly dependent upon experimental procedures, but currently GABA is generally believed to be excitatory only during development and may be restricted to some cortical plate neurons (Ben-Ari, 2014, 2015). In mature neurons, GABA is mainly inhibitory and is generally believed to be excitatory only under certain circumstances when paired with excitatory input (Gulledge and Stuart, 2003).

ChCs in L2/3, on the other hand, have been shown to be capable of mediating excitatory activity in brain slices from animals well past the developmental period when GABA-mediated excitation is thought to occur (Szabadics et al., 2006). The GABA-mediated excitation by ChCs was attributed largely to differences in the intracellular Cl^−^ regulation at the postsynaptic AIS (Szabadics et al., 2006; Khirug et al., 2008; Báldi et al., 2010), a cellular subregion known to be molecularly and physiologically unique (Rasband, 2010; Bender and Trussell, 2012; Kole and Stuart, 2012). To avoid changes in intracellular Cl^−^ concentrations, Szabadics et al. used the gramicidin perforated patch technique (Szabadics et al., 2006). Some subsequent studies in L2/3 using this technique have strengthened their claim (Khirug et al., 2008; Woodruff et al., 2009). However, no direct evidence has been found if this actually occurs *in vivo*. Nevertheless, a possibility for ChC excitation is hypothesized to occur during “down” states, when pyramidal neurons are hyperpolarized and sodium channels are deinactivated (Szabadics et al., 2006; Woodruff et al., 2011).

Because the perforated patch technique may still alter GABAergic activity, concerns with this technique and the results obtained were raised (Glickfeld et al., 2009; Woodruff et al., 2010). Using a novel noninvasive approach that avoids the perturbations with perforated patching, hippocampal ChCs were found to strictly mediate inhibition (Glickfeld et al., 2009; Bazelot et al., 2010; Chiang et al., 2012). Other novel techniques used in the cortex also indicate that ChCs are inhibitory. Using noninvasive methods combined with an innovative technique to activate axo-axonic synapses, Wang et al. (2014) showed that ChCs in the piriform cortex are inhibitory and mediate reversal potentials similar to those mediated by BCs. Noninvasive techniques that replicated *in vivo* conditions indicated that L2/3 ChCs were predominately inhibitory (Woodruff et al., 2011).* In vivo* studies that clearly demonstrate whether ChCs are inhibitory or excitatory are lacking, but some results suggest that ChCs are not excitatory (Klausberger et al., 2003, 2005; Massi et al., 2012; Somogyi et al., 2013; Viney et al., 2013).

ChC-mediated excitation is an enigmatic issue. This is largely due to the complications when recording GABAergic responses. Nevertheless, currently those experiments using the least invasive techniques, along with *in vivo* data, suggest that ChCs are inhibitory, as originally assumed (Somogyi, 1977) and demonstrated above and by others (Buhl et al., 1994; Maccaferri et al., 2000; Tamás and Szabadics, 2004; González-Burgos et al., 2005; Jiang et al., 2013, 2015; Lee et al., 2015).

## ChC Dysfunctions in Brain Disorders

ChC dysfunctions are well associated with schizophrenia (Lewis et al., 2005; Lewis, 2011; Marín, 2012). Evidence indicates the GABA membrane transporter 1 (GAT1) is decreased in axon terminals of ChCs (Woo et al., 1998), whereas the GABA_A_ receptor α2 subunit is increased in pyramidal neurons in schizophrenia (Volk et al., 2002). These pre- and postsynaptic alterations are significantly prominent in L2/3 (Pierri et al., 1999; Volk et al., 2001; Lewis, 2011). The postsynaptic alterations were originally assumed to be compensatory (Volk et al., 2002). If ChCs are excitatory in L2/3, decreased GAT1 may be a compensatory response for decreased excitatory inputs to pyramidal neurons in schizophrenia (Lewis et al., 2012). However, if L2/3 ChCs are inhibitory, then the alterations may be causative or contributory. Future studies will need to examine the effect of these alterations on ChC-mediated GABAergic activity in L2/3.

Schizophrenia is associated with significant changes in neural activity. These changes are shown with both structural and functional alterations and result in abnormal neural network oscillations and synchrony (Meyer-Lindenberg et al., 2001; Uhlhaas and Singer, 2010; Yu et al., 2012). Disruptions in gamma oscillations, which are associated with some of the cognitive dysfunctions in schizophrenia, may result from dysfunctions specifically in ChCs and BCs (Lewis et al., 2012). Schizophrenia patients exhibit many disturbances in cognition, including impairments in attention and sensory processing (Elvevåg and Goldberg, 2000; Javitt, 2009). L2/3 ChCs are part of a cortical interneuronal circuit that is thought to be involved in the selection of attentional and salient signals (Jiang et al., 2013). Dysfunctions of L2/3 ChCs in schizophrenia would then lead to dysfunctions in this circuit, and therefore cause disruptions in attention. The mechanisms underlying schizophrenia are thought to result in part from abnormalities in the development of GABAergic interneuronal circuits (Le Magueresse and Monyer, 2013; Schmidt and Mirnics, 2015). Recent advances in the understanding of ChC development may also lead to hypotheses for how ChC circuitry is altered in schizophrenia. Because the migration of ChCs to L2/3 occurs by P7 when GABA is thought to be excitatory (Anderson and Coulter, 2013; Taniguchi et al., 2013), ChC excitation could have a role in brain maturation and brain disorders (Ben-Ari et al., 2012a).

ChC dysfunction is also implicated in epilepsy (DeFelipe, 1999; Dinocourt et al., 2003). Evidence indicates that ChCs may prevent runaway excitation. ChC axon terminals are lost at the epileptic focus (Ribak, 1985). In addition, *in vivo* recordings show that ChCs fire more robustly than other types of cortical neurons when overall cortical excitation increases (Zhu et al., 2004). Therefore, ChCs may be specifically recruited by epileptic activity to decrease excessive excitation (Paz and Huguenard, 2015). This has led to the hypothesis that ChCs play a critical role in regulating the balance between excitation and inhibition (Zhu et al., 2004). Dysregulation of the excitation-inhibition balance is thought to underlie epilepsy, along with other brain disorders including schizophrenia (Fritschy, 2008; Yizhar et al., 2011; Lewis et al., 2012). Future studies will need to determine the role of ChC dysfunctions in altering the excitation-inhibition balance and whether ChC dysfunctions underlie other brain disorders that implicate disruptions of the excitation-inhibition balance, such as autism (Rubenstein and Merzenich, 2003). Nevertheless, these results suggest that ChCs are at least predominately inhibitory.

## Future Directions

A significant impediment that prevents elucidating the link between ChC dysfunctions and brain disorders is the uncertainties in the role of ChCs in the cortical microcircuit, including whether ChCs are inhibitory or excitatory. Ideally, the ability to specifically target ChCs with the identification of specific markers will greatly aid in resolving the role of ChCs in brain functions. One such undertaking is the creation of the Nkx2.1CreERT2 mouse line that can label a portion of interneurons, the majority of which are ChCs (Taniguchi et al., 2013). With the ability to track ChCs from their genesis to postnatal development and incorporation into cortical circuits, understanding the link between dysfunctions in ChC connectivity and brain disorders may be facilitated (Anderson and Coulter, 2013; Taniguchi et al., 2013). Because of the diversity of ChCs and the possibility of subgroups of ChCs with distinct connectivity patterns and functional roles, innovating techniques will be needed in uncovering ChC circuitry. One such technique is the use of simultaneous multiple patch-clamp recording (Wang et al., 2015; Wyskiel et al., 2016), which can not only greatly elucidate ChC connectivity, but can answer some of the lingering questions about its postsynaptic effects. Further advances in our understanding of ChCs will hopefully provide answers in the near future.

## Conflict of Interest Statement

The authors declare that the research was conducted in the absence of any commercial or financial relationships that could be construed as a potential conflict of interest. The reviewer GGB and handling Editor declared their shared affiliation, and the handling Editor states that the process nevertheless met the standards of a fair and objective review.

## References

[B1] Anderson S. Coulter D. (2013). Neuronal birth to cortical circuitry. Science 340, 1058–1059. 10.1126/science.1235778 23723226

[B2] Bagnall M. W. Hull C. Bushong E. A. Ellisman M. H. Scanziani M. (2011). Multiple clusters of release sites formed by individual thalamic afferents onto cortical interneurons ensure reliable transmission. Neuron 71, 180–194. 10.1016/j.neuron.2011.05.032 21745647 PMC3271052

[B3] Báldi R. Varga C. Tamás G. (2010). Differential distribution of KCC2 along the axo-somato-dendritic axis of hippocampal principal cells. Eur. J. Neurosci. 32, 1319–1325. 10.1111/j.1460-9568.2010.07361.x 20880357

[B4] Baude A. Bleasdale C. Dalezios Y. Somogyi P. Klausberger T. (2007). Immunoreactivity for the GABA_A_ receptor α1 subunit, somatostatin and connexin36 distinguishes axoaxonic, basket and bistratified interneurons of the rat hippocampus. Cereb. Cortex 17, 2094–2107. 10.1093/cercor/bhl117 17122364

[B5] Bazelot M. Dinocourt C. Cohen I. Miles R. (2010). Unitary inhibitory field potentials in the CA_3_ region of rat hippocampus. J. Physiol. 588, 2077–2090. 10.1113/jphysiol.2009.185918 20403979 PMC2911213

[B6] Ben-Ari Y. (2002). Excitatory actions of GABA during development: the nature of the nurture. Nat. Rev. Neurosci. 3, 728–739. 10.1038/nrn920 12209121

[B7] Ben-Ari Y. (2014). The GABA excitatory/inhibitory developmental sequence: a personal journey. Neuroscience 279, 187–219. 10.1016/j.neuroscience.2014.08.001 25168736

[B8] Ben-Ari Y. (2015). Commentary: GABA depolarizes immature neurons and inhibits network activity in the neonatal neocortex in vivo Front. Cell. Neurosci. 9:478 10.3389/fncel.2015.00478 26733806 PMC4686958

[B9] Ben-Ari Y. Cherubini E. Corradetti R. Gaiarsa J. (1989). Giant synaptic potentials in immature rat CA_3_ hippocampal neurones. J. Physiol. 416, 303–325. 10.1113/jphysiol.1989.sp017762 2575165 PMC1189216

[B10] Ben-Ari Y. Khalilov I. Kahle K. T. Cherubini E. (2012a). The GABA excitatory/inhibitory shift in brain maturation and neurological disorders. Neuroscientist 18, 467–486. 10.1177/1073858412438697 22547529

[B11] Ben-Ari Y. Woodin M. A. Sernagor E. Cancedda L. Vinay L. Rivera C.. (2012b). Refuting the challenges of the developmental shift of polarity of GABA actions: GABA more exciting than ever! Front. Cell. Neurosci. 6:35. 10.3389/fncel.2012.00035 22973192 PMC3428604

[B12] Bender K. J. Trussell L. O. (2012). The physiology of the axon initial segment. Annu. Rev. Neurosci. 35, 249–265. 10.1146/annurev-neuro-062111-150339 22443507

[B13] Bennett M. V. Zukin R. S. (2004). Electrical coupling and neuronal synchronization in the mammalian brain. Neuron 41, 495–511. 10.1016/s0896-6273(04)00043-1 14980200

[B14] Blazquez-Llorca L. Woodruff A. Inan M. Anderson S. A. Yuste R. DeFelipe J.. (2015). Spatial distribution of neurons innervated by chandelier cells. Brain Struct. Funct. 220, 2817–2834. 10.1007/s00429-014-0828-3 25056931 PMC4549388

[B15] Bock D. D. Lee W.-C. A. Kerlin A. M. Andermann M. L. Hood G. Wetzel A. W.. (2011). Network anatomy and *in vivo* physiology of visual cortical neurons. Nature 471, 177–182. 10.1038/nature09802 21390124 PMC3095821

[B16] Bregestovski P. Bernard C. (2012). Excitatory GABA: how a correct observation may turn out to be an experimental artifact. Front. Pharmacol. 3:65. 10.3389/fphar.2012.00065 22529813 PMC3329772

[B17] Buhl E. H. Halasy K. Somogyi P. (1994). Diverse sources of hippocampal unitary inhibitory postsynaptic potentials and the number of synaptic release sites. Nature 368, 823–828. 10.1038/368823a0 8159242

[B18] Cherubini E. Gaiarsa J. L. Ben-Ari Y. (1991). GABA: an excitatory transmitter in early postnatal life. Trends Neurosci. 14, 515–519. 10.1016/0166-2236(91)90003-d 1726341

[B19] Chiang P.-H. Wu P.-Y. Kuo T.-W. Liu Y.-C. Chan C.-F. Chien T.-C.. (2012). GABA is depolarizing in hippocampal dentate granule cells of the adolescent and adult rats. J. Neurosci. 32, 62–67. 10.1523/JNEUROSCI.3393-11.2012 22219270 PMC6621339

[B20] Cruz D. A. Eggan S. M. Lewis D. A. (2003). Postnatal development of pre–and postsynaptic GABA markers at chandelier cell connections with pyramidal neurons in monkey prefrontal cortex. J. Comp. Neurol. 465, 385–400. 10.1002/cne.10833 12966563

[B22] De Carlos J. A. López-Mascaraque L. Ramón y Cajal-Agüeras S. Valverde F. (1987). Chandelier cells in the auditory cortex of monkey and man: a Golgi study. Exp. Brain Res. 66, 295–302. 10.1007/bf00243306 3595776

[B21] De Carlos J. A. Lopez-Mascaraque L. Valverde F. (1985). Development, morphology and topography of chandeller cells in the auditory cortex of the cat. Dev. Brain Res. 22, 293–300. 10.1016/0165-3806(85)90182-8 4052819

[B23] DeFelipe J. (1999). Chandelier cells and epilepsy. Brain 122, 1807–1822. 10.1093/brain/122.10.1807 10506085

[B24] DeFelipe J. Hendry S. Jones E. Schmechel D. (1985). Variability in the terminations of GABAergic chandelier cell axons on initial segments of pyramidal cell axons in the monkey sensory–motor cortex. J. Comp. Neurol. 231, 364–384. 10.1002/cne.902310307 2981907

[B25] Del Pino I. García-Frigola C. Dehorter N. Brotons-Mas J. R. Alvarez-Salvado E. Martínez de Lagrán M. M.. (2013). Erbb4 deletion from fast-spiking interneurons causes schizophrenia-like phenotypes. Neuron 79, 1152–1168. 10.1016/j.neuron.2013.07.010 24050403

[B26] Del Río M. R. DeFelipe J. (1994). A study of SMI 32-stained pyramidal cells, parvalbumin-immunoreactive chandelier cells and presumptive thalamocortical axons in the human temproal neocortex. J. Comp. Neurol. 342, 389–408. 10.1002/cne.903420307 7517410

[B27] Dinocourt C. Petanjek Z. Freund T. F. Ben–Ari Y. Esclapez M. (2003). Loss of interneurons innervating pyramidal cell dendrites and axon initial segments in the CA1 region of the hippocampus following pilocarpine–induced seizures. J. Comp. Neurol. 459, 407–425. 10.1002/cne.10622 12687707

[B28] Dugladze T. Schmitz D. Whittington M. A. Vida I. Gloveli T. (2012). Segregation of axonal and somatic activity during fast network oscillations. Science 336, 1458–1461. 10.1126/science.1222017 22700932

[B29] Dzhala V. Valeeva G. Glykys J. Khazipov R. Staley K. (2012). Traumatic alterations in GABA signaling disrupt hippocampal network activity in the developing brain. J. Neurosci. 32, 4017–4031. 10.1523/JNEUROSCI.5139-11.2012 22442068 PMC3333790

[B30] Elvevåg B. Goldberg T. E. (2000). Cognitive impairment in schizophrenia is the core of the disorder. Crit. Rev. Neurobiol. 14, 1–21. 10.1615/critrevneurobiol.v14.i1.10 11253953

[B31] Fairén A. Valverde F. (1980). A specialized type of neuron in the visual cortex of cat: a Golgi and electron microscope study of chandelier cells. J. Comp. Neurol. 194, 761–779. 10.1002/cne.901940405 7204642

[B32] Fariñas I. DeFelipe J. (1991). Patterns of synaptic input on corticocortical and corticothalamic cells in the cat visual cortex. II. The axon initial segment. J. Comp. Neurol. 304, 70–77. 10.1002/cne.903040106 2016413

[B33] Fino E. Yuste R. (2011). Dense inhibitory connectivity in neocortex. Neuron 69, 1188–1203. 10.1016/j.neuron.2011.02.025 21435562 PMC3086675

[B34] Fish K. N. Hoftman G. D. Sheikh W. Kitchens M. Lewis D. A. (2013). Parvalbumin-containing chandelier and basket cell boutons have distinctive modes of maturation in monkey prefrontal cortex. J. Neurosci. 33, 8352–8358. 10.1523/JNEUROSCI.0306-13.2013 23658174 PMC3684962

[B35] Freund T. Martin K. Smith A. Somogyi P. (1983). Glutamate decarboxylase–immunoreactive terminals of Golgi–impregnated axoaxonic cells and of presumed basket cells in synaptic contact with pyramidal neurons of the cat’s visual cortex. J. Comp. Neurol. 221, 263–278. 10.1002/cne.902210303 6655085

[B36] Fritschy J.-M. (2008). Epilepsy, E/I balance and GABA_A_ receptor plasticity. Front. Mol. Neurosci. 1:5. 10.3389/neuro.02.005.2008 18946538 PMC2525999

[B37] Glickfeld L. L. Roberts J. D. Somogyi P. Scanziani M. (2009). Interneurons hyperpolarize pyramidal cells along their entire somatodendritic axis. Nat. Neurosci. 12, 21–23. 10.1038/nn.2230 19029887 PMC3505023

[B38] González-Burgos G. Krimer L. S. Povysheva N. V. Barrionuevo G. Lewis D. A. (2005). Functional properties of fast spiking interneurons and their synaptic connections with pyramidal cells in primate dorsolateral prefrontal cortex. J. Neurophysiol. 93, 942–953. 10.1152/jn.00787.2004 15385591

[B39] Gulledge A. T. Stuart G. J. (2003). Excitatory actions of GABA in the cortex. Neuron 37, 299–309. 10.1016/s0896-6273(02)01146-7 12546824

[B40] Howard A. Tamas G. Soltesz I. (2005). Lighting the chandelier: new vistas for axo-axonic cells. Trends Neurosci. 28, 310–316. 10.1016/j.tins.2005.04.004 15927687

[B41] Inan M. Anderson S. A. (2014). The chandelier cell, form and function. Curr. Opin. Neurobiol. 26, 142–148. 10.1016/j.conb.2014.01.009 24556285 PMC4024324

[B42] Inan M. Blázquez-Llorca L. Merchán-Pérez A. Anderson S. A. DeFelipe J. Yuste R. (2013). Dense and overlapping innervation of pyramidal neurons by chandelier cells. J. Neurosci. 33, 1907–1914. 10.1523/JNEUROSCI.4049-12.2013 23365230 PMC3711719

[B43] Inan M. Welagen J. Anderson S. A. (2012). Spatial and temporal bias in the mitotic origins of somatostatin-and parvalbumin-expressing interneuron subgroups and the chandelier subtype in the medial ganglionic eminence. Cereb. Cortex 22, 820–827. 10.1093/cercor/bhr148 21693785 PMC3450921

[B44] Inda M. DeFelipe J. Muñoz A. (2007). The distribution of chandelier cell axon terminals that express the GABA plasma membrane transporter GAT-1 in the human neocortex. Cereb. Cortex 17, 2060–2071. 10.1093/cercor/bhl114 17099065

[B45] Inda M. DeFelipe J. Muñoz A. (2009). Morphology and distribution of chandelier cell axon terminals in the mouse cerebral cortex and claustroamygdaloid complex. Cereb. Cortex 19, 41–54. 10.1093/cercor/bhn057 18440949

[B46] Javitt D. C. (2009). Sensory processing in schizophrenia: neither simple nor intact. Schizophr. Bull. 35, 1059–1064. 10.1093/schbul/sbp110 19833806 PMC2762632

[B47] Jiang X. Shen S. Cadwell C. R. Berens P. Sinz F. Ecker A. S.. (2015). Principles of connectivity among morphologically defined cell types in adult neocortex. Science 350:aac9462. 10.1126/science.aac9462 26612957 PMC4809866

[B48] Jiang X. Wang G. Lee A. J. Stornetta R. L. Zhu J. J. (2013). The organization of two new cortical interneuronal circuits. Nat. Neurosci. 16, 210–218. 10.1038/nn.3305 23313910 PMC3589105

[B49] Jones E. (1975). Varieties and distribution of non–pyramidal cells in the somatic sensory cortex of the squirrel monkey. J. Comp. Neurol. 160, 205–267. 10.1002/cne.901600204 803518

[B50] Kaila K. (1994). Ionic basis of GABA A receptor channel function in the nervous system. Prog. Neurobiol. 42, 489–537. 10.1016/0301-0082(94)90049-3 7522334

[B51] Kawaguchi Y. (1995). Physiological subgroups of nonpyramidal cells with specific morphological characteristics in layer II/III of rat frontal cortex. J. Neurosci. 15, 2638–2655. 7722619 10.1523/JNEUROSCI.15-04-02638.1995 PMC6577784

[B52] Kawaguchi Y. Kubota Y. (1998). Neurochemical features and synaptic connections of large physiologically-identified GABAergic cells in the rat frontal cortex. Neuroscience 85, 677–701. 10.1016/s0306-4522(97)00685-4 9639265

[B53] Khirug S. Yamada J. Afzalov R. Voipio J. Khiroug L. Kaila K. (2008). GABAergic depolarization of the axon initial segment in cortical principal neurons is caused by the Na-K-2Cl cotransporter NKCC1. J. Neurosci. 28, 4635–4639. 10.1523/JNEUROSCI.0908-08.2008 18448640 PMC6670448

[B54] Kirmse K. Kummer M. Kovalchuk Y. Witte O. W. Garaschuk O. Holthoff K. (2015). GABA depolarizes immature neurons and inhibits network activity in the neonatal neocortex *in vivo*. Nat. Commun. 6:7750. 10.3389/fphar.2015.00294 26177896

[B55] Kisvárday Z. Adams C. Smith A. (1986). Synaptic connections of axo-axonic (chandelier) cells in human epileptic temporal cortex. Neuroscience 19, 1179–1186. 10.1016/0306-4522(86)90131-4 3029627

[B56] Klausberger T. Magill P. J. Márton L. F. Roberts J. D. B. Cobden P. M. Buzsáki G.. (2003). Brain-state-and cell-type-specific firing of hippocampal interneurons *in vivo*. Nature 421, 844–848. 10.1038/nature01374 12594513

[B57] Klausberger T. Marton L. F. O’Neill J. Huck J. H. Dalezios Y. Fuentealba P.. (2005). Complementary roles of cholecystokinin-and parvalbumin-expressing GABAergic neurons in hippocampal network oscillations. J. Neurosci. 25, 9782–9793. 10.1523/JNEUROSCI.3269-05.2005 16237182 PMC6725722

[B58] Kole M. H. Stuart G. J. (2012). Signal processing in the axon initial segment. Neuron 73, 235–247. 10.1016/j.neuron.2012.01.007 22284179

[B59] Kosaka T. (1983). Axon initial segments of the granule cell in the rat dentate gyrus: synaptic contacts on bundles of axon initial segments. Brain Res. 274, 129–134. 10.1016/0006-8993(83)90527-9 6616249

[B61] Lee A. J. Wang G. Jiang X. Johnson S. M. Hoang E. T. Lanté F.. (2015). Canonical organization of layer 1 neuron-led cortical inhibitory and disinhibitory interneuronal circuits. Cereb. Cortex 25, 2114–2126. 10.1093/cercor/bhu020 24554728 PMC4494026

[B60] Le Magueresse C. Monyer H. (2013). GABAergic interneurons shape the functional maturation of the cortex. Neuron 77, 388–405. 10.1016/j.neuron.2013.01.011 23395369

[B62] Lewis D. A. (2011). The chandelier neuron in schizophrenia. Dev. Neurobiol. 71, 118–127. 10.1002/dneu.20825 21154915 PMC3116957

[B64] Lewis D. A. Curley A. A. Glausier J. R. Volk D. W. (2012). Cortical parvalbumin interneurons and cognitive dysfunction in schizophrenia. Trends Neurosci. 35, 57–67. 10.1016/j.tins.2011.10.004 22154068 PMC3253230

[B65] Lewis D. A. Hashimoto T. Volk D. W. (2005). Cortical inhibitory neurons and schizophrenia. Nat. Rev. Neurosci. 6, 312–324. 10.1038/nrn1648 15803162

[B63] Lewis D. A. Lund J. S. (1990). Heterogeneity of chandelier neurons in monkey neocortex: corticotropin–releasing factor–and parvalbumin–immunoreactive populations. J. Comp. Neurol. 293, 599–615. 10.1002/cne.902930406 2329196

[B66] Li X.-G. Somogyi P. Tepper J. Buzsáki G. (1992). Axonal and dendritic arborization of an intracellularly labeled chandelier cell in the CA1 region of rat hippocampus. Exp. Brain Res. 90, 519–525. 10.1007/bf00230934 1385200

[B67] Liu B.-H. Li P. Li Y.-T. Sun Y. J. Yanagawa Y. Obata K.. (2009). Visual receptive field structure of cortical inhibitory neurons revealed by two-photon imaging guided recording. J. Neurosci. 29, 10520–10532. 10.1523/JNEUROSCI.1915-09.2009 19710305 PMC2779138

[B68] Loebel A. Silberberg G. Helbig D. Markram H. Tsodyks M. Richardson M. J. (2009). Multiquantal release underlies the distribution of synaptic efficacies in the neocortex. Front. Comput. Neurosci. 3:27. 10.3389/neuro.10.027.2009 19956403 PMC2786302

[B69] Maccaferri G. Roberts J. D. Szucs P. Cottingham C. A. Somogyi P. (2000). Cell surface domain specific postsynaptic currents evoked by identified GABAergic neurones in rat hippocampus *in vitro*. J. Physiol. 524, 91–116. 10.1111/j.1469-7793.2000.t01-3-00091.x 10747186 PMC2269850

[B70] Marín O. (2012). Interneuron dysfunction in psychiatric disorders. Nat. Rev. Neurosci. 13, 107–120. 10.1038/nrn3155 22251963

[B71] Marin-Padilla M. (1987). The chandelier cell of the human visual cortex: a Golgi study. J. Comp. Neurol. 256, 61–70. 10.1002/cne.902560106 2434536

[B72] Markram H. Muller E. Ramaswamy S. Reimann M. W. Abdellah M. Sanchez C. A.. (2015). Reconstruction and simulation of neocortical microcircuitry. Cell 163, 456–492. 10.1016/j.cell.2015.09.029 26451489

[B73] Massi L. Lagler M. Hartwich K. Borhegyi Z. Somogyi P. Klausberger T. (2012). Temporal dynamics of parvalbumin-expressing axo-axonic and basket cells in the rat medial prefrontal cortex *in vivo*. J. Neurosci. 32, 16496–16502. 10.1523/JNEUROSCI.3475-12.2012 23152631 PMC4487822

[B74] Meyer-Lindenberg A. Poline J.-B. Kohn P. D. Holt J. L. Egan M. F. Weinberger D. R.. (2001). Evidence for abnormal cortical functional connectivity during working memory in schizophrenia. Am. J. Psychiatry 158, 1809–1817. 10.1176/appi.ajp.158.11.1809 11691686

[B75] Misgeld U. Deisz R. Dodt H. Lux H. (1986). The role of chloride transport in postsynaptic inhibition of hippocampal neurons. Science 232, 1413–1415. 10.1126/science.2424084 2424084

[B76] Mueller A. L. Taube J. S. Schwartzkroin P. A. (1984). Development of hyperpolarizing inhibitory postsynaptic potentials and hyperpolarizing response to gamma-aminobutyric acid in rabbit hippocampus studied *in vitro*. J. Neurosci. 4, 860–867. 6707735 10.1523/JNEUROSCI.04-03-00860.1984 PMC6564832

[B77] Niell C. M. Stryker M. P. (2008). Highly selective receptive fields in mouse visual cortex. J. Neurosci. 28, 7520–7536. 10.1523/JNEUROSCI.0623-08.2008 18650330 PMC3040721

[B78] Obata K. Oide M. Tanaka H. (1978). Excitatory and inhibitory actions of GABA and glycine on embryonic chick spinal neurons in culture. Brain Res. 144, 179–184. 10.1016/0006-8993(78)90447-x 638760

[B80] Owens D. F. Boyce L. H. Davis M. B. Kriegstein A. R. (1996). Excitatory GABA responses in embryonic and neonatal cortical slices demonstrated by gramicidin perforated-patch recordings and calcium imaging. J. Neurosci. 16, 6414–6423. 8815920 10.1523/JNEUROSCI.16-20-06414.1996 PMC6578913

[B79] Owens D. F. Kriegstein A. R. (2002). Is there more to GABA than synaptic inhibition? Nat. Rev. Neurosci. 3, 715–727. 10.1038/nrn919 12209120

[B81] Packer A. M. McConnell D. J. Fino E. Yuste R. (2013). Axo-dendritic overlap and laminar projection can explain interneuron connectivity to pyramidal cells. Cereb. Cortex 23, 2790–2802. 10.1093/cercor/bhs210 22941716 PMC3968298

[B82] Paz J. T. Huguenard J. R. (2015). Microcircuits and their interactions in epilepsy: is the focus out of focus? Nat. Neurosci. 18, 351–359. 10.1038/nn.3950 25710837 PMC4561622

[B83] Peters A. Proskauer C. C. Ribak C. E. (1982). Chandelier cells in rat visual cortex. J. Comp. Neurol. 206, 397–416. 10.1002/cne.902060408 7096634

[B84] Pierri J. N. Chaudry A. S. Woo T.-U.W. Lewis D. A. (1999). Alterations in chandelier neuron axon terminals in the prefrontal cortex of schizophrenic subjects. Am. J. Psychiatry 156, 1709–1719. 10553733 10.1176/ajp.156.11.1709

[B85] Rasband M. N. (2010). The axon initial segment and the maintenance of neuronal polarity. Nat. Rev. Neurosci. 11, 552–562. 10.1038/nrn2852 20631711

[B86] Ribak C. E. (1985). Axon terminals of GABAergic chandelier cells are lost at epileptic foci. Brain Res. 326, 251–260. 10.1016/0006-8993(85)90034-4 2982461

[B87] Rivera C. Voipio J. Payne J. A. Ruusuvuori E. Lahtinen H. Lamsa K.. (1999). The K^+^/Cl^−^ co-transporter KCC2 renders GABA hyperpolarizing during neuronal maturation. Nature 397, 251–255. 10.1038/16697 9930699

[B88] Rubenstein J. Merzenich M. (2003). Model of autism: increased ratio of excitation/inhibition in key neural systems. Genes Brain Behav. 2, 255–267. 10.1034/j.1601-183x.2003.00037.x 14606691 PMC6748642

[B89] Schmidt M. J. Mirnics K. (2015). Neurodevelopment, GABA system dysfunction and schizophrenia. Neuropsychopharmacology 40, 190–206. 10.1038/npp.2014.95 24759129 PMC4262918

[B90] Sloper J. Powell T. (1979). A study of the axon initial segment and proximal axon of neurons in the primate motor and somatic sensory cortices. Philos. Trans. R. Soc. Lond. B Biol. Sci. 285, 173–197. 10.1098/rstb.1979.0004 88058

[B91] Sohya K. Kameyama K. Yanagawa Y. Obata K. Tsumoto T. (2007). GABAergic neurons are less selective to stimulus orientation than excitatory neurons in layer II/III of visual cortex, as revealed by *in vivo* functional Ca^2+^ imaging in transgenic mice. J. Neurosci. 27, 2145–2149. 10.1523/jneurosci.4641-06.2007 17314309 PMC6673543

[B92] Somogyi P. (1977). A specific ‘axo-axonal’interneuron in the visual cortex of the rat. Brain Res. 136, 345–350. 10.1016/0006-8993(77)90808-3 922488

[B93] Somogyi P. Freund T. Cowey A. (1982). The axo-axonic interneuron in the cerebral cortex of the rat, cat and monkey. Neuroscience 7, 2577–2607. 10.1016/0306-4522(82)90086-0 7155343

[B94] Somogyi P. Freund T. F. Hodgson A. J. Somogyi J. Beroukas D. Chubb I. W. (1985). Identified axo-axonic cells are immunoreactive for GABA in the hippocampus visual cortex of the cat. Brain Res. 332, 143–149. 10.1016/0006-8993(85)90397-x 3995258

[B95] Somogyi P. Katona L. Klausberger T. Lasztóczi B. Viney T. J. (2013). Temporal redistribution of inhibition over neuronal subcellular domains underlies state-dependent rhythmic change of excitability in the hippocampus. Philos. Trans. R. Soc. Lond. B Biol. Sci. 369:20120518. 10.1098/rstb.2012.0518 24366131 PMC3866441

[B96] Somogyi P. Nunzi M. Gorio A. Smith A. (1983). A new type of specific interneuron in the monkey hippocampus forming synapses exclusively with the axon initial segments of pyramidal cells. Brain Res. 259, 137–142. 10.1016/0006-8993(83)91076-4 6824927

[B97] Szabadics J. Varga C. Molnár G. Oláh S. Barzó P. Tamas G. (2006). Excitatory effect of GABAergic axo-axonic cells in cortical microcircuits. Science 311, 233–235. 10.1126/science.1121325 16410524

[B98] Szentágothai J. (1975). The ‘module-concept’ in cerebral cortex architecture. Brain Res. 95, 475–496. 10.1016/0006-8993(75)90122-5 808252

[B99] Szentágothai J. Arbib M. A. (1974). Conceptual models of neural organization. Neurosci. Res. Program. Bull. 12, 305–510. 4437759

[B100] Tai Y. Janas J. A. Wang C.-L. Van Aelst L. (2014). Regulation of chandelier cell cartridge and bouton development via DOCK7-mediated ErbB4 activation. Cell Rep. 6, 254–263. 10.1016/j.celrep.2013.12.034 24440718 PMC3920736

[B101] Tamás G. Szabadics J. (2004). Summation of unitary IPSPs elicited by identified axo-axonic interneurons. Cereb. Cortex 14, 823–826. 10.1093/cercor/bhh051 15084495

[B102] Taniguchi H. Lu J. Huang Z. J. (2013). The spatial and temporal origin of chandelier cells in mouse neocortex. Science 339, 70–74. 10.1126/science.1227622 23180771 PMC4017638

[B103] Uhlhaas P. J. Singer W. (2010). Abnormal neural oscillations and synchrony in schizophrenia. Nat. Rev. Neurosci. 11, 100–113. 10.1038/nrn2774 20087360

[B104] Varga C. Lee S. Y. Soltesz I. (2010). Target-selective GABAergic control of entorhinal cortex output. Nat. Neurosci. 13, 822–824. 10.1038/nn.2570 20512133 PMC3139425

[B105] Viney T. J. Lasztoczi B. Katona L. Crump M. G. Tukker J. J. Klausberger T.. (2013). Network state-dependent inhibition of identified hippocampal CA3 axo-axonic cells *in vivo*. Nat. Neurosci. 16, 1802–1811. 10.1038/nn.3550 24141313 PMC4471148

[B106] Volk D. W. Austin M. C. Pierri J. N. Sampson A. R. Lewis D. A. (2001). GABA transporter-1 mRNA in the prefrontal cortex in schizophrenia: decreased expression in a subset of neurons. Am. J. Psychiatry 158, 256–265. 10.1176/appi.ajp.158.2.256 11156808

[B107] Volk D. W. Pierri J. N. Fritschy J.-M. Auh S. Sampson A. R. Lewis D. A. (2002). Reciprocal alterations in pre-and postsynaptic inhibitory markers at chandelier cell inputs to pyramidal neurons in schizophrenia. Cereb. Cortex 12, 1063–1070. 10.1093/cercor/12.10.1063 12217970

[B110] Wang X. Hooks B. M. Sun Q.-Q. (2014). Thorough GABAergic innervation of the entire axon initial segment revealed by an optogenetic ‘laserspritzer’. J. Physiol. 592, 4257–4276. 10.1113/jphysiol.2014.275719 25085892 PMC4215776

[B109] Wang X. Sun Q.-Q. (2012). Characterization of axo-axonic synapses in the piriform cortex of Mus musculus. J. Comp. Neurol. 520, 832–847. 10.1002/cne.22792 22020781 PMC3903392

[B108] Wang G. Wyskiel D. R. Yang W. Wang Y. Milbern L. C. Lalanne T.. (2015). An optogenetics-and imaging-assisted simultaneous multiple patch-clamp recording system for decoding complex neural circuits. Nat. Protoc. 10, 397–412. 10.1038/nprot.2015.019 25654757 PMC4505930

[B111] Woo T.-U. Whitehead R. E. Melchitzky D. S. Lewis D. A. (1998). A subclass of prefrontal γ-aminobutyric acid axon terminals are selectively altered in schizophrenia. Proc. Natl. Acad. Sci. U S A 95, 5341–5346. 10.1093/cercor/8.7.614 9560277 PMC20262

[B112] Woodruff A. Xu Q. Anderson S. A. Yuste R. (2009). Depolarizing effect of neocortical chandelier neurons. Front. Neural Circuits 3:15. 10.3389/neuro.04.015.2009 19876404 PMC2769545

[B113] Woodruff A. R. Anderson S. A. Yuste R. (2010). The enigmatic function of chandelier cells. Front. Neurosci. 4:201. 10.3389/fnins.2010.00201 21151823 PMC2999891

[B114] Woodruff A. R. McGarry L. M. Vogels T. P. Inan M. Anderson S. A. Yuste R. (2011). State-dependent function of neocortical chandelier cells. J. Neurosci. 31, 17872–17886. 10.1523/JNEUROSCI.3894-11.2011 22159102 PMC4071969

[B115] Wyskiel D. R. Larry T. C. Jiang X. Wang G. Zhu J. J. (2016). Analysis of transsynaptic attentional neuronal circuits with octuple patch-clamp recordings. Adv. Patch Clamp Anal. Neurosci. 113, 139–150. 10.1007/978-1-4939-3411-9_7

[B116] Xu X. Callaway E. M. (2009). Laminar specificity of functional input to distinct types of inhibitory cortical neurons. J. Neurosci. 29, 70–85. 10.1523/JNEUROSCI.4104-08.2009 19129386 PMC2656387

[B117] Yizhar O. Fenno L. E. Prigge M. Schneider F. Davidson T. J. O’Shea D. J.. (2011). Neocortical excitation/inhibition balance in information processing and social dysfunction. Nature 477, 171–178. 10.1038/nature10360 21796121 PMC4155501

[B118] Yu Q. Allen E. A. Sui J. Arbabshirani M. R. Pearlson G. Calhoun V. D. (2012). Brain connectivity networks in schizophrenia underlying resting state functional magnetic resonance imaging. Curr. Top. Med. Chem. 12, 2415–2425. 10.2174/156802612805289890 23279180 PMC4429862

[B119] Zhu Y. Stornetta R. L. Zhu J. J. (2004). Chandelier cells control excessive cortical excitation: characteristics of whisker-evoked synaptic responses of layer 2/3 nonpyramidal and pyramidal neurons. J. Neurosci. 24, 5101–5108. 10.1523/jneurosci.0544-04.2004 15175379 PMC6729194

